# Utility of Patient-Reported Symptom and Functional Outcomes to Indicate Recovery after First 90 Days of Radical Cystectomy: A Longitudinal Study

**DOI:** 10.3390/cancers15113051

**Published:** 2023-06-04

**Authors:** Xin Shelley Wang, Kelly K. Bree, Neema Navai, Mona Kamal, Shu-En Shen, Elizabeth Letona, Charles S. Cleeland, Qiuling Shi, Vijaya Gottumukkala

**Affiliations:** 1 Department of Symptom Research, The University of Texas MD Anderson Cancer Center, Houston, TX 77030, USA; mkjomaa@mdanderson.org (M.K.); sshen@mdanderson.org (S.-E.S.); egonzalez11@mdanderson.org (E.L.); cscleeland@gmail.com (C.S.C.); 2 Department of Urology, The University of Texas MD Anderson Cancer Center, Houston, TX 77030, USA; kbree@mdanderson.org (K.K.B.); nnavai@mdanderson.org (N.N.); 3 School of Public Health and Management, Chongqing Medical University, Chongqing 400016, China; qshi@cqmu.edu.cn; 4 Department of Anesthesiology & Perioperative Medicine, The University of Texas MD Anderson Cancer Center, Houston, TX 77030, USA; vgottumukkala@mdanderson.org

**Keywords:** postoperative symptoms, functional recovery, patient-reported outcomes (PROs), perioperative care, MD Anderson Symptom Inventory (MDASI), cystectomy

## Abstract

**Simple Summary:**

One barrier to implementing patient-reported outcomes (PROs) during perioperative care for bladder cancer (BLC) patients is the lack of empirical reports of meaningful symptom burden that are associated with postoperative recovery. This study aimed to describe symptom burden and functioning status for 3 months post-radical cystectomy, using a validated disease-specific PRO measure tool, the MD Anderson Symptom Inventory (the MDASI-PeriOp-BLC). We found that the most severe symptom burden at baseline and discharge is associated with poor functional recovery post-radical cystectomy for BLC. These PROs could be used to identify BLC patients at the highest risk for poor functional recovery during the perioperative period. We also found that postoperative functional recovery assessment via PROs is more feasible than an objective performance measure. The completion of MDASI-PeriOp-BLC at preoperative, discharge and end of study was 100%, 79% and 77%, while Timed Up and Go test completion rates were 88%, 54% and 13%, respectively.

**Abstract:**

This is a longitudinal prospective study that tracked multiple symptom burden and functioning status for bladder cancer (BLC) patients for 3 months post-radical cystectomy at The University of Texas MD Anderson Cancer Center, using a validated disease-specific patient-reported outcome measure (PROM) tool, the MD Anderson Symptom Inventory (the MDASI-PeriOp-BLC). The feasibility of collecting an objective measure for physical functioning, using “Timed Up & Go test” (TUGT) and PRO scores at baseline, discharge and end of study, was tested. Patients (*n* = 52) received care under an ERAS pathway. The more severe scores of fatigue, sleep disturbance, distress, drowsiness, frequent urination and urinary urgency at baseline predicted poor functional recovery postoperatively (OR = 1.661, 1.039–2.655, *p* = 0.034); other more severe symptoms at discharge (pain, fatigue, sleep disturbance, lack of appetite, drowsiness, bloating/abdominal tightness) predicted poor functional recovery (OR = 1.697, 1.114–2.584, *p* = 0.014) postoperatively. Compliance rates at preoperative, discharge and end of study were 100%, 79% and 77%, while TUGT completion rates were 88%, 54% and 13%, respectively. This prospective study found that more severe symptom burden at baseline and discharge is associated with poor functional recovery post-radical cystectomy for BLC. The collection of PROs is more feasible than using performance measures (TUGT) of function following radical cystectomy.

## 1. Introduction

Radical cystectomy, with or without neoadjuvant chemotherapy, is a curative treatment option for recurrent non-muscle invasive or locally advanced stages and a palliative treatment option for the metastatic disease of bladder cancer (BLC) [1,2,3]. With improved surgical care in patients with cancer, there is increased attention to using patient-reported outcomes (PROs) in perioperative care to improve clinical outcomes [4]. Although the awareness of the role of PROs in many clinical settings is growing, PRO research in BLC postoperative care has not been fully explored.

A systematic review found that only eight BLC randomized clinical trials included PROs between 2014 and 2018, and the quality of reported PROs was found to be inadequate [5]. PROs have been more widely and effectively utilized in other malignancies. PROs have been used as a measure of functional recovery post-thoracic surgery for lung cancer [6] and found to be more feasible to collect compared to objective performance measures in the postoperative setting [7].

While uniform reporting standards for PRO use in BLC are important to fully utilize such data [5], disease/treatment-specific PRO tools are also critical. The MD Anderson Symptom Inventory (MDASI-PeriOpBLC) has been specifically developed and psychometrically validated for assessing symptom burden and functioning in the perioperative period for BLC patients [8], and this was utilized in the current study.

Complications after radical cystectomy occur in 54–80% of patients during the first 90 days after surgery [9,10,11]. In addition to the typical postoperative symptom burden, such as postoperative pain and fatigue experienced by many surgical patients [12], radical cystectomy represents unique functional recovery challenges [10]. Longitudinally monitoring perioperative symptom burden and functional outcomes might help symptom management in a timely manner, accelerating postoperative recovery, increasing patient satisfaction, and decreasing postoperative complications. To date, there has been relatively little progress in terms of utilization, assessment and understanding of PROs as outcomes in routine postoperative care for BLC [5,13].

To better characterize the role of PROs in the perioperative care of patients with BLC, we conducted a prospective longitudinal study to document patient-reported symptoms and daily functioning before and in the first 90 days after radical cystectomy for BLC. Using a psychometrically validated perioperative version of the MD Anderson Symptom Inventory, MDASI-PeriOp-BLC, we sought to identify patients at the highest risk for poor functional recovery during the perioperative period. We also compared the feasibility of postoperative function recovery assessment via both PROs and an objective performance measure using the “Timed Up & Go test” (TUGT).

## 2. Materials and Methods

### 2.1. Participants

Eligible patients included those at least 18 years old who spoke English, had a diagnosis of BLC and been scheduled for radical cystectomy with curative or palliative intent, had no diagnosis of active psychosis or severe cognitive impairment, understood the study’s intent, and were willing to participate. This study was approved by the Institutional Review Board, and written informed consent was obtained from all patients.

### 2.2. Data Collection and PRO Measurement

Eligible BLC patients were enrolled during the preoperative clinic visit at MD Anderson between October 2018 and July 2021. PRO measures were either completed online by the patients or over the phone by a trained study coordinator. In this longitudinal study, we utilized REDCap [14] as the data collection platform to administer the validated MDASI-PeriOp-BLC module (8) and to track electronic PROs (ePROs) perioperatively, daily during hospitalization, on the day of discharge, post-discharge on days 3 and 7, weekly during first 12 weeks after surgery and at the end of the study.

The study coordinator assessed the patients’ performance status at the initial assessment using the Eastern Cooperative Oncology Group (ECOG) performance status scale [15], and clinical, demographic and pathological data were collected via chart review. The presence of comorbid conditions according to the Charlson Comorbidity Index [16] was also collected.

The symptom assessment tool MDASI-PeriOp-BLC [8] was developed and psychometrically validated for use in perioperative care for patients with BLC, followed with FDA guidance for development and validation of PRO tools [17]. MDASI-PeriOp-BLC includes 13 common cancer-related symptoms (fatigue, pain, sleep disturbance, poor appetite, distress, drowsiness, dry mouth, shortness of breath, sadness, numbness/tingling, nausea, vomiting, difficulty remembering) and 8 PeriOp-BLC module items (including blood in urine, leaking urine, frequent urination, urinary urgency, constipation, burning with urination, changes in sexual function and stomal problems). Functional status measurement included 6 symptom interferences items (general activity, mood, walk, work, relation with others and enjoyment of life). Patients rated the severity of the symptoms they experienced on a 0–10 numeric rating scale, with 0 meaning no symptom and 10 meaning “as bad as you can imagine”. The recall period for measuring symptom severity and functional interferences of the MDASI was the previous 24 h. The cognitive debriefing assessed ease of completion, comprehensibility, acceptability, redundancy, use of the scoring system, item clarification and content domain confirmation of the new instrument.

### 2.3. Objective Physical Functioning Measure

The objective-timed performance test (TUGT) [18,19] measures the time it takes a participant to rise from a chair, walk 3 m, turn, walk back and sit down again. TUGT, as a performance outcome measurement tool, was assessed preoperatively, by discharge day, and at the first postoperative outpatient follow-up visit. A final score was recorded as the mean score of two attempts of TUGT at each time point and was categorized into 3 groups (normal (≤10 s), frail (11–20 s) and prolonged (needs further evaluation) (>20 s)), based on the literature [18,19].

### 2.4. Statistical Analysis

Descriptive statistics for patients and clinical information were reported as mean, standard deviation, median, minimum and maximum for continuous variables and percent for categorical variables. Loess curves present the most severe symptoms over time during the first 90 days after discharge. We defined the prevalence of moderate symptoms and composite scores of interferences as 5–6 on a 0–10 scale, and severe as ≥7 on a 0–10 scale on MDASI-PeriOp-BLC. A composite score selection was based on the 6 items with the highest mean severity score before surgery or at discharge. Composite scores were calculated as the mean of selected items.

An average score of a cluster of the most severe symptoms, both before surgery and at discharge, was calculated. Group-based trajectory modeling (GBTM) [20,21] was used to identify distinct functional recovery trajectories over time with individual PRO items on MDASI Interference [6] and the average score. We generated a two-group model with the goal of simplicity and clinical interpretability that represents two memberships, either high or low symptom burden, over the time period. Individuals with persistently reported high symptom scores are placed in the high-trajectory group, whereas those with persistently low symptom scores are placed in the low-trajectory group. SAS macro PROC TRAJ [22] was used to estimate the trajectory of the MDASI total interference, physical functioning (WAW: work, general activity, walk) and psychological functioning (REM: relation with others, enjoyment of life and mood) scores, using all data collected from discharge to the end of the study. 

The strength of the association between the average score of the top severe symptoms before surgery or at discharge and the group of patients with persistent high interference scores (outcome variable) was estimated via multinomial logistic regression models. We used odds ratios to measure the magnitude of the severity of the average score (as a continuous variable) as predictive of poor recovery on patient’s daily functioning (composite score of total interferences, physical functioning and psychological functioning) after discharge to 90 days. The covariance in the regression modeling included age (65+ vs. <65 yrs old), CCI (2+ vs. 0–1) and receipt of neoadjuvant chemotherapy (yes vs. no). The association of recovery membership with end of study QoL and end of study health status separately was calculated using paired-sample *t*-tests.

All statistical procedures were performed using SAS statistical software, version 9.4 (SAS Institute, Cary, NC, USA). All *p* values reported are 2-tailed. Statistical significance was set at *p* < 0.05.

## 3. Results

### 3.1. Participants

A total of 52 patients were enrolled in this longitudinal study and were included in the analysis. Of those patients, two patients withdrew from the study at days 3 and 7, and other patients contributed PROs for the first 90 days (median 89 days, range 32–108 days). Demographic and disease-related characteristics are summarized in Table 1. Enrolled patients were predominantly older adults (mean age 66.5 years old, standard deviation (SD) (9.30)), male (87%) and white (94%). Most of the patients had ECOG performance status of 0–1 (90%), and 27 (52%) had a Charlson Comorbidity Index score of less than or equal to 2. Further, 52% (*n* = 27) of patients undergoing radical cystectomy had muscle-invasive BLC, while 48% (*n* = 25) had non-muscle invasive disease. With respect to type of urinary diversion, the majority of patients were treated with an ileal conduit (77%), followed by neobladder (21%) and continent urinary reservoir (2%). The majority (67%) of patients received neoadjuvant chemotherapy, and all received care under an ERAS pathway [23]. The median length of stay was 6 days (IQR: 5–8 days).

### 3.2. Application of MDASI-PeriOp-BLC

#### Patient Compliance

The items with the highest level of missing values post-operatively were frequent urination (9%), urinary urgency (8%), leaking urine (8%), pain or burning with urination (8%) and blood in urine (4%).

Severity and prevalence of symptom burden, health status and quality of life at pre-surgery, discharge and end of study were included.

**Figure 1:**
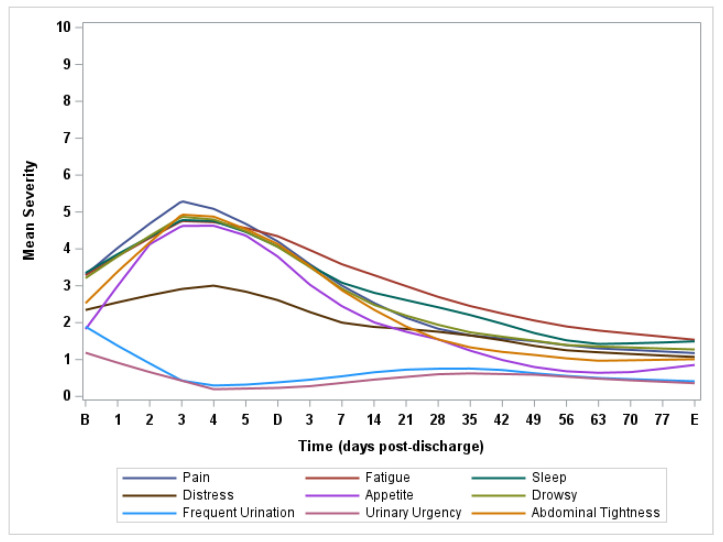
Lowess curves of major symptom burden post-radical cystectomy on MDASI-PeriOp-BLC. B = Baseline. D = Discharge. E = End of Study.

Figure 1 presents the natural history of perioperative symptom burden in the first 90 days after bladder surgery on MDASI-PeriOp-BLC.

Table 2 presents the severity and prevalence of moderate to severe MDASI-PeriOp-BLC symptoms and composite scores of interferences at baseline, discharge and end of study. It also presents the severity of the single-item quality-of-life (SIQOL) question and EQ-5D visual analogue scale (VAS) score to indicate generic health status and quality of life. At baseline, the most severe symptoms were fatigue, disturbed sleep, distress, drowsiness, frequent urination and urinary urgency. At discharge (*n* = 41), the most severe symptoms were pain, fatigue, disturbed sleep, lack of appetite, drowsiness and bloating/abdominal tightness. At the end of the study (*n* = 40), an average of 89 days after surgery, few patients reported moderate to severe symptoms. Although we observed significant worsening of multiple general symptom burden items (on MDASI-core symptom items) on discharge day than preoperative (as expected in the immediate postoperative period), there was significant recovery of bladder-cancer-related symptoms on MDASI-PeriOp-BLC module items 90 days post-operatively, with most patients having better MDASI-PeriOp-BLC scores at the end of the study than they did pre-operatively. 

Table 2 also presents that 37% of patients reported moderate to severe levels of functional interferences at 5 or greater, or 7 or greater on the total scores of MDASI Interferences (general activity, mood, work, walking, relations with others and enjoyment of life) at discharge, which was much higher than pre-surgery (11.5%) and 90-days post-surgery (5.5%).

### 3.3. Trajectory Membership of Postoperative Symptom Functioning Recovery

**Figure 2:**
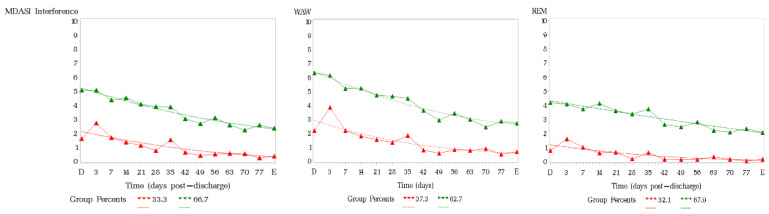
Functioning recovery trend by trajectory of membership of MDASI-Interferences. D = Discharge. E = End of Study.

Figure 2 presents the results from trajectory analysis; a group of 66.7% of patients reported persistently higher symptom interferences over time post-surgery on the MDASI total interference composite score.

The group with high interference scores had a significant association with patient-reported health status on EQ5D5L (but not with SIQOL) at the first postoperative follow-up (*n* = 36, mean 41.83 days post-surgery) in the domains of usual activities (*p* < 0.0001), self-care (*p* = 0.0015), pain/discomfort (*p* = 0.0337) and VAS of health status (*p* = 0.001).

### 3.4. Impact of Symptom Severity on Postoperative Function Recovery

Regression analysis in Table 3 shows that the composite score of the six most severe symptoms at baseline (disturbed sleep, fatigue, distress, drowsiness, frequent urination, urinary urgency) were significant predictors of the interference symptom trajectory group in the univariate model (OR 1.661, 95% CI 1.039–2.655, *p* = 0.0339). Table 3 also presents the composite scores of the six most severe symptoms at discharge (fatigue, pain, disturbed sleep, lack of appetite, drowsiness and bloating/abdominal tightness) that were significantly associated with high symptom trajectory group membership in the multivariate model (OR 1.697, 95% CI 1.114–2.584, *p* = 0.014). Both models were controlled for age, CCI and neoadjuvant chemotherapy status.

### 3.5. Compliance with Objective and Subjective Measures of Physical Functioning

For the functional assessment, the data available for both PROs on MDASI Interferences and TUGT were recorded. Compliance rates at preoperative, discharge and end of study of MDASI Interferences were 100%, 79% and 77%, while TUGT completion rates were 88%, 54% and 13%, respectively.

Most patients at the baseline and end of study time points did not complete the TUGT test because they completed PRO assessments remotely but were not able to perform TUGT. At the discharge time point, most of the patients who did not complete the TUGT test declined to complete the test.

Spearman correlation showed that the severity of the patient-reported “Walking” interference item on MDASI-I was significantly associated with prolonged TUGT score at baseline (r = 0.3256, *p* = 0.0273).

## 4. Discussion

This longitudinal study demonstrated the feasibility of utilizing PROs for measuring both symptom and functional outcomes via the MDASI-PeriOp-BLC module, before and during the first 90 days after radical cystectomy. The current study demonstrated that more severe symptom burden, both at baseline and discharge, is predictive of poor functional recovery after radical cystectomy for BLC. However, our study also highlights that despite severe symptom scores in the pre- and immediate postoperative setting, by 3 months post-operatively, many patients had improved QOL and functional status compared to their baseline. Compared to the objective-timed performance test on physical functioning measure (TUGT), completion of PROs (MDASI Interference) was more feasible for monitoring functional status in the BLC postoperative setting.

PRO data are essential to implement personalized treatment plans after cystectomy and can be helpful in predicting outcomes and treatment side effects. Somani et al. [24] previously showed no difference in QoL of patients pre- and post-cystectomy with neobladders, using European Organization for Research and Treatment of Cancer QLQ-C30 and Satisfaction With Life Scale. In a study assessing quality of life (QOL) in BLC patients, two-thirds of the respondents reported at least one HRQOL problem, with mobility issues being most commonly reported, followed by pain and discomfort [25]. Further, in a study among patients undergoing GYN surgery, we reported critical disease-/treatment-specific symptoms (abdominal bloating and cramping) at discharge that were found to be significantly relevant to assessing the risk of grade 2 or higher complications 30 days post-laparotomy [26]. In the current study, we elucidated the recovery characteristics by using a validated PROM tool, MDASI-Periop-BLC, defining the symptom cluster at critical timepoints that are clinically meaningful during the postoperative period after cystectomy. Nevertheless, as shown in Table 2 and Table 3, frequent urination and urinary urgency before cystectomy, and the post-cystectomy symptoms of bloating/abdominal tightness at discharge, are time-sensitive and clinically meaningful to determine the surgical recovery, while other major general symptom burden on MDASI core items (pain, fatigue, sleep disturbance, drowsiness) is also critical to include for monitoring and identifying individuals who might experience a less-ideal functioning recovery journey 3 months after surgery.

Measuring functional recovery is an important part of postoperative care. Our study indicated that 37% of patients reported moderate to severe levels of symptom interference at discharge (based upon 5 or greater on MDASI Interferences), although with a trend of improvement over time. As a marker of impaired recovery, we found that MDADI Interference scores were significantly related to multiple domains of health status on EQ5D5L at the first postoperative follow-up clinic visit. As with our earlier reports in patients undergoing thoracic and abdominal procedures [6,7,26], this study adds validity to the use of the interference item subscale of MDASI (MDASI-I) as a PRO functional measure in postoperative care of patients’ post-radical cystectomy.

In a previous study among patients undergoing laparotomy for gynecologic tumors [7], we reported that MDASI-I could be used as a surrogate or potential substitution for TUGT to predict patient’s physical functioning, both pre- and postoperatively. Consistent with our previous impressions from the gynecologic surgery cohort, this study again confirms that the subjective functioning measure via PROs is a valid and much easier measure of functional status than an in-person objective measure with TUGT. Similar to other studies on TUGT, in which prolonged time was associated with the need for physical assistance [27,28,29], in this study, we found that the severity of the patient-reported “Walking” interference item on MDASI-I was significantly associated with a prolonged TUGT score at baseline (r = 0.3256, *p* = 0.0273).

Currently, increased research supports patient empowerment through the integration of real-time PRO monitoring in postoperative care. For high-risk individuals, PRO assessment coupled with triage responsive interventions have the potential to improve the quality of perioperative symptom management in cancer patients [30,31] and enhance functional recovery after surgery [6,32,33,34,35,36]. Our study provides evidence to support future studies on the effectiveness of PROs after radical cystectomy to improve functioning recovery in this cohort of patients.

This study has several limitations. This real-world study is a single-institution study performed at a large tertiary care cancer center, where the majority of the patients are non-Hispanic White males. A further study should be conducted with a more diverse patient population to confirm our findings. Additionally, nearly half of the patients undergoing radical cystectomy in this cohort underwent surgery for non-muscle invasive disease. These patients had very high-risk non-muscle invasive disease, often associated with lymphovascular invasion, variant histology, etc., and many received neoadjuvant chemotherapy. We expect that the use of PROs in the assessment of functional recovery after extirpative surgery is equally as effective in these patients as their muscle-invasive counterparts. Finally, the pre-selected cutoff points for the presented moderate to severe symptoms at 5+ and 7+ on a 0–10 scale of severity were not determined individually, which should be confirmed in a future study for their clinical meaningfulness in BLC postoperative care.

## 5. Conclusions

In conclusion, the monitoring of perioperative symptoms and function using PROs is feasible during the perioperative period after radical cystectomy. Functional status assessment using PROs correlated with functional performance, as measured by more time- and resource-intensive objective measurements such as the TUGT. The impact of routine PRO monitoring on improving postoperative recovery warrants further investigation.

## Figures and Tables

**Table 1:** Baseline demographic and clinical characteristics (*n* = 52).

Patient Characteristics	Mean (SD)	Median (Range)
Age, years	66.52 (9.3)	66.62 (39.71–86.39)
	**Frequency**	**Percent**
Age Group		
<65	22	42.31
≥65	30	57.69
Sex		
Men	45	86.54
Women	7	13.46
Race		
White	49	94.23
Others	3	5.77
Ethnicity		
Hispanic or Latino	2	3.85
Not Hispanic	50	96.15
Marital Status		
Married/Partnered	41	78.85
Single/Others	11	21.15
Education		
High School	11	21.15
College	34	65.38
Graduate/Professional training	7	13.46
Job status		
Employed outside the home	21	40.38
Homemaker/Retired	26	50
Medical leave/disable due to illness	5	9.62
ECOG		
Missing	2	3.85
Good (0–1)	47	90.38
Poor (2–3)	3	5.77
Charlson Comorbidity Index		
0	2	3.85
1–2	25	48.08
3–4	18	34.62
5–7	7	13.46
Bladder Cancer Stage (Tumor)		
Ta	2	3.85
Tis	5	9.62
T1	20	38.46
T2	21	40.38
T3	4	7.69
Bladder Cancer Stage (Node)		
N0	49	94.23
N1	3	5.77
Bladder Cancer Stage (Metastasis)		
M0	51	98.08
M1	1	1.92
Under ERAS Pathway		
No	1	1.92
Yes	51	98.08
Intraoperative Complications		
No	52	100
Yes	0	0
Postoperative Complications		
No	26	50
Yes	26	50
Recurrent disease		
No	42	80.77
Yes	10	19.23
Neoadjuvant chemotherapy		
No	17	32.69
Yes	35	67.31

**Table 2:** Symptom severity and prevalence of moderate to severe symptoms on MDASI-Periop-BLC, subscales and health status of EQ5D and quality-of-life measure at baseline, discharge and end of study.

	Baseline				Discharge (6 Days Post-Surgery ± 2 Days)				End of Study (89 Days Post-Surgery ± 2.5 Days)			
	*n*	Mean (SD)	% ≥5	% ≥7	*n*	Mean (SD)	% ≥5	% ≥7	*n*	Mean (SD)	% ≥5	% ≥7
**Single Item Quality of Life**	52	7.04 (2.82)	86.54	67.31	43	6.19 (2.39)	81.40	46.51	39	5.82 (3.01)	69.23	53.85
EQ-5D VAS Score *^,^†	52	74.33 (22.55)	-	-	43	65.40 (16.19)	-	-	39	78.08 (18.15)	-	-
**MDASI-Core Symptom Items**												
Pain *	52	0.83 (1.45)	3.85	0	**41**	**3.51 (2.49)**	**29.27**	**17.07**	40	1.45 (2.02)	10	5
Fatigue *	**52**	**1.98 (2.40)**	**15.38**	**5.77**	**41**	**3.54 (2.38)**	**34.15**	**9.76**	40	1.63 (1.85)	12.5	0
Nausea *	51	0.35 (1.25)	1.92	1.92	41	1.37 (2.22)	9.76	7.32	40	0.43 (1.43)	2.5	2.5
Disturbed Sleep	**51**	**2.45 (3.01)**	**28.85**	**13.46**	**41**	**3.54 (3.09)**	**31.71**	**21.95**	40	1.78 (2.12)	15	2.5
Distress	**52**	**1.83 (2.17)**	**15.38**	**5.77**	41	2.10 (2.31)	21.95	4.88	40	1.20 (1.84)	10	2.5
Shortness of Breath	52	0.71 (1.40)	1.92	1.92	41	1.02 (1.60)	7.32	0	40	0.75 (1.63)	5	2.5
Difficulty Remembering	52	1.27 (1.73)	3.85	1.92	41	1.41 (2.40)	14.63	7.32	40	1.08 (1.59)	2.5	0
Lack of Appetite *	51	0.88 (1.44)	5.77	0	**41**	**3.66 (2.79)**	**41.46**	**17.07**	40	0.93 (1.61)	5	0
Drowsiness *	**52**	**1.60 (2.36)**	**13.46**	**5.77**	**41**	**3.56 (2.66)**	**34.15**	**9.76**	40	1.18 (1.41)	2.5	0
Dry Mouth *	52	1.13 (2.11)	11.54	3.85	41	2.80 (2.97(	24.39	14.63	40	0.73 (1.15)	2.5	0
Sadness	52	1.42 (2.15)	15.38	1.92	41	1.83 (2.57)	19.51	7.32	40	1.10 (1.58)	2.5	2.5
Vomiting	52	0.19 (0.99)	1.92	1.92	41	0.44 (1.38)	4.88	2.44	40	0.08 (0.27)	0	0
Numbness	52	1.02 (1.84)	5.77	3.85	41	0.90 (1.61)	7.32	0	40	1.00 (1.72)	1	0
**MDASI-PeriOpBLC Module Items**												
Blood in Your Urine †	52	0.96 (2.42)	7.69	5.77	37	1.57 (2.57)	17.07	7.32	39	0.08 (0.27)	0	0
Frequent Urination *^,^†	**52**	**2.83 (2.99)**	**25**	**13.46**	36	0.50 (1.46)	7.32	0	39	0.51 (1.43)	5	0
Leaking Urine *^,^†	52	1.44 (2.81)	13.46	11.54	36	0.47 (1.44)	4.88	0	39	0.44 (0.94)	0	0
Pain or Burning with Urination *^,^†	52	0.85 (1.66)	7.69	0	36	0.03 (0.17)	0	0	38	0.03 (0.16)	0	0
Urinary Urgency *^,^†	**52**	**2.42 (2.97)**	**23.08**	**11.54**	36	0.14 (0.54)	0	0	39	0.49 (1.32)	5	0
Constipation	52	1.04 (1.97)	7.69	1.92	41	1.90 (2.50)	19.51	9.76	40	1.15 (1.83)	12.5	0
Diarrhea *	52	0.56 (1.61)	3.85	1.92	40	1.83 (3.04)	17.07	12.2	40	0.40 (1.41)	2.5	0
Bloating/Abdominal Tightness *^,^†	51	0.33 (0.99)	1.92	0	41	**3.10 (2.64)**	**24.39**	**12.2**	**39**	1.23 (1.72)	7.5	0
Stomal Problems	51	0.25 (0.87)	1.92	0	40	0.63 (1.21)	2.44	0	40	0.35 (0.66)	0	0
**MDASI-Interference Items**												
Walking *	52	1.17 (2.26)	9.62	5.77	40	3.13 (2.67)	31.71	12.2	40	1.45 (1.97)	10	2.5
General Activity *	52	2.19 (3.32)	23.08	17.31	41	5.49 (3.3)	60.98	43.9	40	2.10 (2.28)	22.5	5
Working *	52	1.58 (2.64)	19.23	5.77	40	5.00 (4.19)	51.22	48.78	40	2.20 (2.21)	15	5
Relations with Others *	52	0.88 (2.05)	9.62	1.92	41	2.02 (2.44)	19.51	7.32	40	0.93 (1.53)	7.5	0
Enjoyment of Life *	52	1.73 (2.47)	19.23	3.85	41	3.80 (3.44)	41.46	26.83	40	1.88 (2.22)	17.5	5
Mood *	51	1.76 (2.45)	15.38	5.77	40	2.90 (2.82)	29.27	12.2	40	1.40 (1.75)	15	0
**MDASI Composite Scores**												
Core *	52	1.20 (1.16)	0	0	41	2.28 (1.51)	7.32	0	40	1.02 (1.02)	0	0
Module †	52	1.19 (1.49)	1.92	0	41	1.30 (1.19)	2.44	0	40	0.52 (0.59)	0	0
Interference *	52	1.56 (2.23)	11.54	3.85	41	3.73 (2.47)	36.59	14.63	40	1.66 (1.64)	7.5	0
WAW *	52	1.65 (2.51)	15.38	5.77	41	4.59 (2.94)	53.66	24.39	40	1.92 (1.85)	5	0
REM *	52	1.46 (2.11)	9.62	1.92	41	2.89 (2.45)	21.95	9.76	40	1.40 (1.67)	10	0
Most severe 6 Symptoms at Baseline †	52	2.22 (2.14)	13.46	5.77	41	2.17 (1.61)	7.32	0	40	1.07 (1.26)	0	0
Most severe 6 Symptoms at Discharge *	52	1.34 (1.41)	1.92	0	41	3.48 (2.11)	24.39	4.88	40	1.36 (1.43)	2.50	0

The most severe 6 symptoms at baseline composite score includes fatigue, disturbed sleep, distress, drowsiness, frequent urination, and urinary urgency. The most severe 6 symptoms at discharge composite score includes pain, fatigue, disturbed sleep, lack of appetite, drowsiness, and bloating/abdominal tightness. Bolded rows indicate top symptoms at given timepoint. * Significant difference in mean between baseline and discharge. † Significant difference in mean between end of study and baseline.

**Table 3:** Multivariate analysis of the composite score of most severe symptoms at preoperation and by discharge predicting slow post-operative functioning recovery on MDASI Interference trajectory group membership.

Outcomes on MDASI	Effect	Odds Ratio	95% CI	*p*-Value
High vs. Low membership of total Interference Composite	Severity of Top 6 Baseline Composite ^a^ (0–10 scale)	**1.661**	**1.039–2.655**	**0.0339**
	CCI (>2 vs. ≤2)	0.353	0.056–2.246	0.2701
	Age (≥65 vs. <65)	1.504	0.244–9.255	0.6600
	Received Neoadjuvant Chemotherapy (Yes vs. No)	1.598	0.410–6.235	0.4998
High vs. Low membership of total Interference Composite	Severity of Top 6 Discharge Composite ^b^ (0–10 scale)	**1.697**	**1.114–2.584**	**0.0137**
	CCI (>2 vs. ≤2)	1.369	0.157–11.960	0.7766
	Age (≥65 vs. <65)	0.564	0.064–4.939	0.6052
	Received Neoadjuvant Chemotherapy (Yes vs. No)	1.233	0.272–5.596	0.7863
High vs. Low membership of WAW ^c^ Composite	Top 6 Baseline Composite ^a^	**1.494**	**1.001–2.230**	**0.0494**
	CCI (>2 vs. ≤2)	**0.148**	**0.023–0.940**	**0.0428**
	Age (≥65 vs. <65)	1.858	0.316–10.909	0.4928
	Received Neoadjuvant Chemotherapy (Yes vs. No)	1.102	0.282–4.302	0.8891
High vs. Low membership of WAW ^c^ Composite	Top 6 Discharge Composite ^b^	**1.654**	**1.093–2.503**	**0.0172**
	CCI (>2 vs. ≤2)	0.316	0.043–2.336	0.2592
	Age (≥65 vs. <65)	0.839	0.117–6.032	0.8611
	Received Neoadjuvant Chemotherapy (Yes vs. No)	0.628	0.130–3.036	0.5631
High vs. Low membership of REM ^d^ Composite	Severity of Top 6 Baseline Composite ^a^ (0–10 scale)	**1.617**	**1.025–2.552**	**0.0388**
	CCI (>2 vs. ≤2)	0.451	0.076–2.691	0.3822
	Age (≥65 vs. <65)	1.864	0.323–10.744	0.4861
	Received Neoadjuvant Chemotherapy (Yes vs. No)	1.044	0.266–4.092	0.9511
High vs. Low membership of REM ^d^ Composite	Severity of Top 6 Discharge Composite ^b^ (0–10 scale)	**1.573**	**1.061–2.331**	**0.0242**
	CCI (>2 vs. ≤2)	1.681	0.219–12.883	0.6171
	Age (≥65 vs. <65)	0.726	0.095–5.553	0.7577
	Received Neoadjuvant Chemotherapy (Yes vs. No)	0.799	0.181–3.534	0.7676

^a^ Top 6 symptoms at baseline include disturbed sleep, fatigue, distress, drowsiness, frequent urination, and urinary urgency. ^b^ Top 6 symptoms at discharge include lack of appetite, drowsiness, fatigue, disturbed sleep, pain, and bloated/abdominal tightness. ^c^ WAW includes walking, general activity, and working. ^d^ REM includes relations with others, enjoyment of life, and mood.

## Data Availability

Any data needed to evaluate the conclusions in the paper are present within the paper. Please contact with corresponding author if a further question for research data, which will be considered under institutional ethics policy.
